# Reliability of a wireless instrumented insole (WalkinSense system) for measuring spatiotemporal and kinematic gait variables

**DOI:** 10.1002/jeo2.70628

**Published:** 2026-01-22

**Authors:** Melanie Eckelt, Jennifer Fayad, Anne Backes, Frederic Garcia, Bernd Grimm, Valeria Serchi, Tobias Meyer, Thomas Solignac, Caroline Mouton, Romain Seil, Laurent Malisoux

**Affiliations:** ^1^ Department of Precision Health Luxembourg Institute of Health Strassen Luxembourg; ^2^ IEE S.A. Bissen Luxembourg; ^3^ Department of Orthopaedic Surgery Centre Hospitalier Luxembourg—Clinique d'Eich Eich Luxembourg

**Keywords:** gait analysis, instrumented insoles, test–retest reliability, wearable sensors

## Abstract

**Purpose:**

Reliable gait analysis is essential for clinical assessment and research. Wearable technologies such as the WalkinSense system (WSS), a wireless instrumented insole system equipped with an inertial measurement unit, enable the measurement of spatiotemporal and kinematic gait parameters in real‐world settings. The accuracy of the WSS has been previously validated. This study aims to evaluate the test–retest reliability of the WSS in healthy adults walking and running under various speed and slope conditions.

**Methods:**

Forty‐nine healthy adults completed two sessions one week apart, walking and running at 3, 4.5 (−3°, −6°, +3°, +6°), 6 and 9 km/h on a treadmill. Spatiotemporal variables, including stance time, swing time, stride time, stride length, single and double support time, as well as kinematic variables such as foot ground angle at initial contact and toe‐off, were recorded using the WSS. Relative reliability was assessed using intraclass correlation coefficients (ICC_2,1_), while absolute reliability was evaluated using the standard error of measurement (SEM), minimal detectable change (MDC) and their percentage values (SEM%, MDC%). Bland‐Altman plots were used to detect systematic bias and visualise agreement.

**Results:**

Results demonstrated good to excellent reliability for most spatiotemporal parameters across all conditions, with ICC values ranging from 0.76–0.95, while the foot ground angles exhibited lower reliability (ICC: 0.71–0.86). SEM% and MDC% were generally below 10% for spatiotemporal measures (SEM%: 1.63–6.63; MDC%: 3.44–18.36), reflecting both low measurement error and high sensitivity to detect real changes beyond random variation. Bland‐Altman analyses revealed no relevant heteroscedasticity.

**Conclusion:**

These findings support the WSS as a reliable tool for assessing spatiotemporal variables in healthy adults across diverse walking and running conditions, underscoring its potential for use in both clinical and research environments. However, clinical studies are needed to fully establish its utility in patient assessment.

**Level of Evidence:**

Level II, diagnostic studies.

**Abbreviations**
CI: confidence interval
FGA: foot ground angle
HD‐FSR: high dynamic force sensing resistor
IC: initial contact
ICC: intraclass correlation coefficient
IMU: inertial measurement units
IQR: interquartile range
MDC: minimal detectable change
SD: standard deviation
SEM: standard error of measurement
TO: toe‐off
WMS: within‐subject error variance
WSS: WalkinSense system

## INTRODUCTION

Gait analysis is a well‐established method for quantifying human locomotion and is widely used in sports science, biomechanical research and clinical applications [18, 41]. In orthopaedics, the objective assessment of gait parameters has proven to be a valuable tool for diagnostics, therapy optimisation and monitoring rehabilitation progress [3, 38]. Gold‐standard methods such as motion capture systems, force plates and instrumented treadmills provide precise gait analysis, but are costly, resource‐intensive and restricted to laboratory environments, limiting their applicability in clinical practice and daily life [28].

In rehabilitation, reliable gait assessment is essential for tracking patient progress and evaluating the effects of therapeutic interventions [5, 27, 31]. Wearable sensor technologies, such as instrumented insoles, have been developed as a promising alternative to usual laboratory equipment by enabling gait analysis in real‐life conditions [4].

The WalkinSense System (WSS; IEE S.A.), a wireless instrumented insole system, integrates pressure‐sensitive insoles with inertial measurement unit (IMU), utilising printed force‐sensitive resistor technology to maintain a thin, flexible design that preserves natural gait mechanics and shoe comfort [13, 17]. Compared to many wearable gait analysis systems that rely exclusively on either inertial or pressure sensing, the WSS combines both modalities within a lightweight, shoe‐integrated design. This enables the direct detection of gait events while simultaneously providing kinematic information, which is advantageous for comprehensive gait assessment in real‐world and clinical environments. Although a variety of wearable gait analysis systems exist, the WSS is a relatively new device for which comprehensive scientific evaluation had previously been lacking, particularly regarding validity and test–retest reliability. While gait analysis tools must meet rigorous scientific quality standards, many wearable systems lack comprehensive validation studies [26, 29]. Beyond validation, assessing the reliability of gait metrics is crucial to ensure consistent and reproducible results over repeated measurements [7, 10, 34]. Reliability refers to the consistency and reproducibility of a measurement under identical conditions, ensuring that observed variability primarily reflects true differences rather than measurement error [1]. In the context of gait analysis, high reliability is crucial to differentiate between actual biomechanical changes and fluctuations due to instrument limitations, providing confidence in clinical decision‐making and research findings [20]. Two key components of reliability are relative reliability, typically assessed using the intraclass correlation coefficient (ICC) and absolute reliability, which considers the magnitude of measurement error through metrics like the standard error of measurement (SEM) or the minimal detectable change (MDC) [37]. These metrics are essential for interpreting changes in gait variables, ensuring that observed differences exceed the threshold of measurement error and reflect genuine physiological changes [24].

The accuracy of the WSS has already been validated against a gold‐standard measurement method (i.e., instrumented treadmill and motion capture system) in a recent study [9], which demonstrated good‐to‐excellent validity for key gait variables. Building upon this established validity, the objective of this study was to evaluate the reliability of the WSS by analysing the consistency and agreement of gait metrics across repeated trials. The focus was on spatiotemporal and kinematic variables, as they are commonly used as reference values for detecting gait abnormalities and monitoring rehabilitation outcomes [15, 16, 23]. We hypothesised that reliability is better for spatiotemporal variables, especially stride time, stride length and stance time, compared to the kinematic variables, given the excellent accuracy of these variables previously reported [9]. Determining the reliability of these variables is essential to ensure the robustness of gait assessments and increase the clinical utility of the WSS [30, 36].

## METHODS

### Participants

A total of 52 healthy participants, with no history of lower limb surgery or orthopaedic conditions, volunteered to participate in this study (39% female, mean age 34.8 ± 10.6 years, height 175 ± 10 cm, body mass 74.2 ± 15.6 kg). The study received approval from the National Research Ethics Committee (CNER, approval ref: 202212/04 Version 2.0). All participants were provided with a detailed description of the protocol and gave informed consent before participation. Demographic information, including age, height and body mass, was collected during the first of two testing sessions.

### Instruments

**Figure 1:**
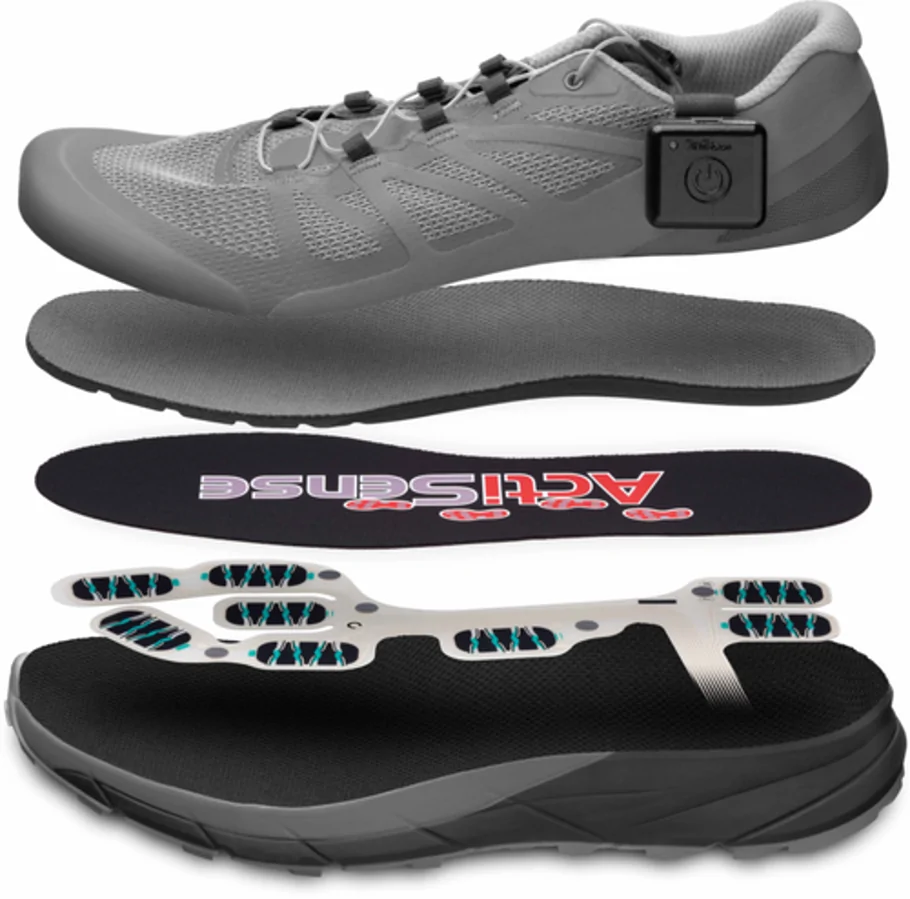
The WalkinSense System (IEE S.A.).

The WSS consists of a hardware setup that includes both an IMU (ICM‐20948) and pressure‐sensitive insoles for data acquisition (Figure 1). The insoles are equipped with eight HD‐FSR (high dynamic force sensing resistor) sensors each, positioned according to established standards for plantar pressure sensor placement and size [29]. The WSS hardware is compact and lightweight, minimizing interference with gait. The IMU can be attached laterally to a wide variety of shoes using a clip (Figure 1). The insoles, with a thickness of 0.35 mm, can be seamlessly integrated into various types of footwear, allowing patients to use their own personal shoes. The WSS offers an extended battery life of up to 10 days of continuous 24‐h use and operates at a sampling frequency up to 200 Hz.

The WSS system also features a digital environment, allowing for Bluetooth control and monitoring of the hardware (recording/streaming, sensors connectivity, battery status, etc.); and raw‐data storage and processing.

All walking and running tests were conducted on a split‐belt treadmill equipped with force plates (sampling frequency of 1 kHz, M‐Gait, Motek Forcelink). However, force‐plate data were not used in this study, as the analysis focused on the reliability of WSS data.

### Data collection

Each participant was fitted with the appropriate WSS insoles based on their shoe size. Participants wore their own familiar sports shoes during data collection. First, participants were instructed to walk on the treadmill at a slow speed of 3 km/h with no slope to familiarize themselves with the experimental setup. Subsequently, they completed a series of nine trials. Each trial consisted of a 1‐min habituation phase followed by 1 min of data recording. The trials were conducted in a fixed, predetermined order: walking at slow speed (3 km/h) with no slope, walking at medium speed (4.5 km/h) with no slope, then with positive slopes of 3° and 6° and negative slopes of −3° and −6°, walking at high speed (6 km/h) with no slope and running (9 km/h) with no slope. These speeds and slope conditions were selected because they represent commonly used treadmill settings in clinical and research practice and cover a realistic range of gait demands. The test and retest sessions followed the same fixed sequence of trials one week apart, ensuring that any potential order effects were consistent across sessions and therefore did not bias the reliability estimates.

### Data processing and statistical analysis

The WSS sampling frequency was set to 100 Hz, and data were resampled to 200 Hz (cubic interpolation). The gyroscope and accelerometer were set up with a full‐scale range of ± 1000 dps and ± 8 G, respectively. Details on data processing and the validation of the system against a gold standard have been published previously [9]. Briefly, custom algorithms were used to calculate the different spatiotemporal and kinematic variables, such as stance time, swing time, stride time, stride length, single support time, double support time, foot ground angle at initial contact (FGA at IC) and foot ground angle at toe off (FGA at TO). The gait variables were calculated for each gait cycle and then averaged per person over the total number of steps taken during the 1‐min data recording.

To identify extreme outliers, the interquartile range (IQR) method was applied. This approach has been used in previous validation and reliability studies of insole‐based sensor systems to ensure data quality and reduce the influence of implausible measurements [39]. The excluded data showed extreme deviations from the group distribution and were not attributable to identifiable procedural errors. Briefly, the distribution of the test–retest differences was examined for each gait variable. Participants were excluded only if their difference values exceeded the IQR‐derived thresholds for the majority of variables, suggesting systematic inconsistencies across conditions. These cases reflected implausible and inconsistent data patterns rather than isolated missing values.

Relative reliability was assessed using the intraclass correlation coefficient model 2,1 (ICC_2,1_) from the R package ‘irr’. A 95% confidence interval (95% CI) was calculated for each parameter to provide an estimate of uncertainty. ICCs were interpreted as poor (< 0.50), moderate (0.50–0.75), good (0.75–0.90), or excellent (≥ 0.90) [20].

Absolute reliability was evaluated using Bland‐Altman plots and by calculating the SEM and MDC. Systematic changes in the mean were examined through Bland‐Altman analysis [11]. The Bland‐Altman plots were created by plotting the difference between sessions against the mean value of the two sessions for each variable. These plots, along with the 95% CI for the test–retest differences, were used to visualize systematic variations relative to the zero line. The 95% CI was determined as the test–retest difference ± 1.96 times the standard error of the test–retest difference.

Measurement errors were assessed using the SEM and SEM%. The SEM was determined by calculating the square root of the within‐subject error variance (WMS), which was estimated from the variance of the differences between two repeated measurements (SD Diff):

$$\text{SEM}=\left(\frac{\text{SD Diff}}{\sqrt{2}}\right)=\sqrt{\mathrm{WMS}}.$$

The SEM%, representing the within‐subject SEMs expressed as a percentage of the mean, was defined as

$$\mathrm{SEM}\%=\left(\frac{\mathrm{SEM}}{\mathrm{Mean}}\right)\times 100.$$

The MDC was calculated as

$$\text{MDC}=\mathrm{SEM}\times \sqrt{2}\times 1.96.$$

To calculate MDC independent of the units of measurement, the MDC% was defined as

$$\mathrm{MDC}\%=\left(\frac{\mathrm{MDC}}{\mathrm{Mean}}\right)\times 100.$$

SEM% values were categorised as indicating low measurement error (SEM% ≤ 10%) or high measurement error (SEM% > 10%), while MDC% values were categorized as indicating low MDC (MDC% ≤ 20%), acceptable MDC (20% < MDC% < 40%) or high MDC (MDC% ≥ 40%) [2, 35].

A sensitivity analysis was conducted to assess the effect of the step count (i.e., size of the data recording used) on reliability statistics (MDC%), comparing results based on all analysed steps (Table 1) with those derived from the first 10, 20 and 30 steps performed. All statistical analyses were conducted in R using RStudio (V.4.3.2).

**Table 1:** Number of participants, average and SD of the total step count for all test and retest conditions (speeds and slopes).

Condition	Participants	Total step count	
(km/h; °)	*n*	Test (mean ± SD)	Retest (mean ± SD)
3; 0	47	89.23 ± 10.55	88.84 ± 8.38
4.5; 0	48	105.69 ± 9.07	106.65 ± 6.46
4.5; −3	48	106.48 ± 10.95	106.61 ± 9.85
4.5; −6	48	108.69 ± 9.93	110.00 ± 6.49
4.5; 3	48	102.43 ± 11.55	105.02 ± 7.33
4.5; 6	47	102.58 ± 10.01	103.06 ± 8.78
6; 0	49	115.92 ± 11.51	117.10 ± 11.31
9; 0	49	154.10 ± 11.03	155.43 ± 8.85

Abbreviation: SD, standard deviation.

## RESULTS

Of the 52 participants tested, three were excluded due to the IQR method. Due to missing or inaccurate data (e.g., noisy signals), some test conditions (speeds and slopes) had fewer participants. The number of participants per condition, the average and standard deviation (SD) of the step counts across test and retest conditions are shown in Table 1.

**Figure 2:**
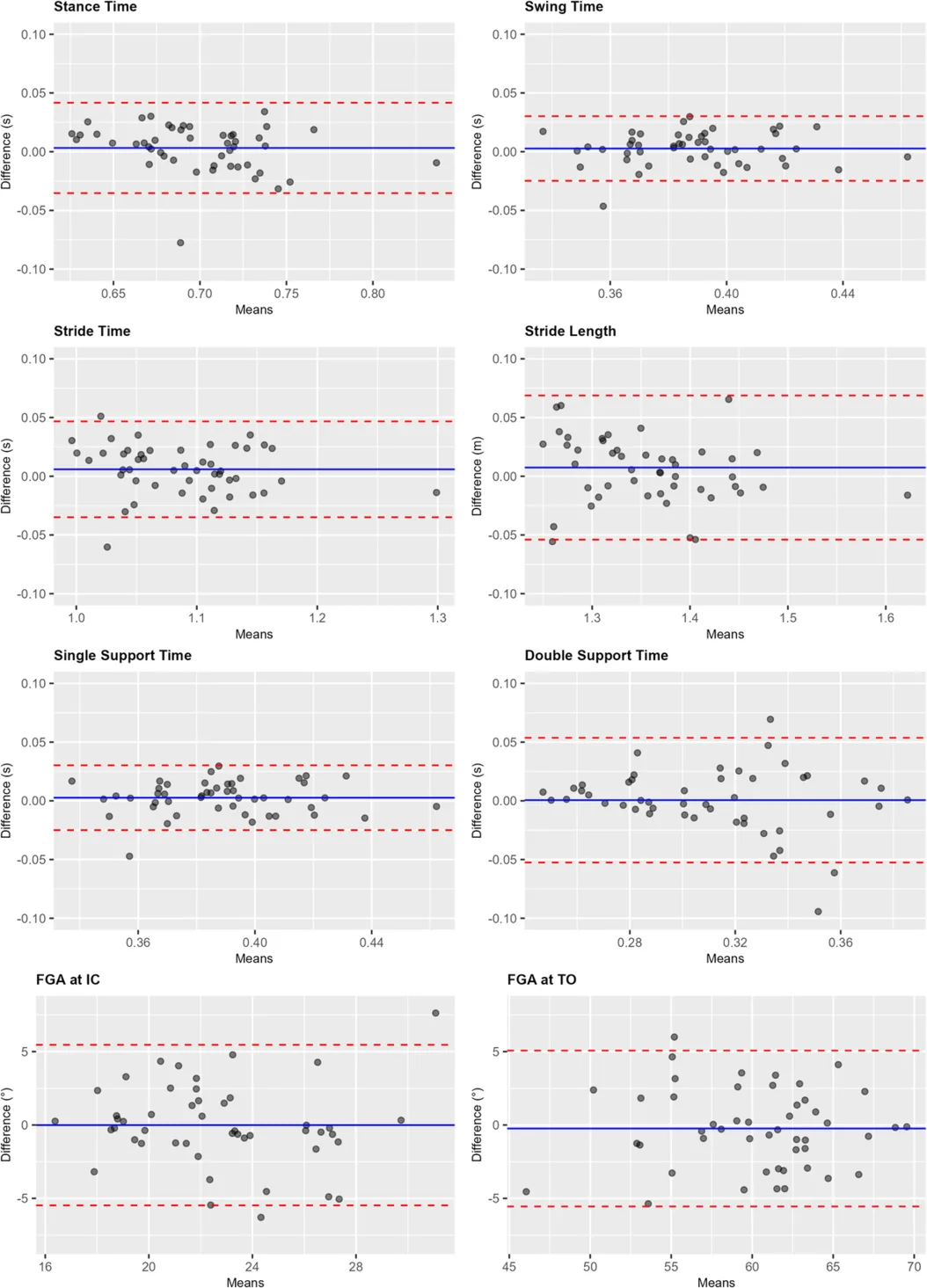
Bland‐Altman plots for different gait variables at 4.5 km/h (no slope). FGA, foot ground angle; IC, initial contact; TO, toe‐off.

Figure 2 presents Bland‐Altman plots for the eight gait variables at 4.5 km/h (no slope). The Bland‐Altman plots for the other conditions (speeds and slopes) are available in the Supporting Information S1. Across all variables, mean differences were close to zero, indicating no systematic bias between tests and retests. Data points were generally evenly distributed around the mean difference, with very few outliers for some of the variables, indicating consistent agreement. However, variability differs among variables, with FGA at IC and FGA at TO showing the largest differences and the widest limits of agreement, suggesting greater measurement variability compared to spatiotemporal gait variables. These observations were consistent across the different speed and slope conditions (see Supporting Information S1).

Table 2 shows descriptive and reliability statistics for the spatiotemporal and kinematic gait variables across different conditions (speeds and slopes). For each condition, the mean and SD of test and retest measurements are reported alongside the ICC 2,1 (with 95% CI), SEM (with SEM%) and MDC (with MDC%).

**Table 2:** Descriptive statistics of test and retest, ICC, SEM and MDC values for all test conditions (speeds and slopes).

Condition (km/h;°)	Test (mean ± SD)	Retest (mean ± SD)	ICC_2,1_ (95% CI)	SEM (%)	MDC (%)
Stance time (s)					
3; 0	0.840 ± 0.066	0.857 ± 0.058	0.82 (0.65–0.91)	0.024 (2.85)	0.067 (7.90)
4.5; 0	0.698 ± 0.044	0.701 ± 0.038	0.89 (0.81–0.93)	0.014 (1.98)	0.038 (5.50)
4.5; −3	0.685 ± 0.045	0.686 ± 0.036	0.87 (0.78–0.93)	0.017 (2.36)	0.047 (6.53)
4.5; −6	0.669 ± 0.047	0.669 ± 0.040	0.88 (0.80–0.93)	0.016 (2.20)	0.044 (6.09)
4.5; 3	0.711 ± 0.050	0.713 ± 0.043	0.85 (0.75–0.92)	0.016 (2.29)	0.044 (6.34)
4.5; 6	0.717 ± 0.048	0.720 ± 0.043	0.90 (0.83–0.94)	0.014 (2.11)	0.039 (5.85)
6; 0	0.617 ± 0.038	0.619 ± 0.036	0.93 (0.87–0.96)	0.010 (1.65)	0.028 (4.56)
9; 0	0.343 ± 0.037	0.344 ± 0.038	0.87 (0.78–0.94)	0.014 (3.95)	0.038 (10.94)
Swing time (s)					
3; 0	0.439 ± 0.040	0.443 ± 0.040	0.82 (0.70–0.89)	0.017 (3.84)	0.047 (10.66)
4.5; 0	0.388 ± 0.026	0.390 ± 0.027	0.86 (0.76–0.92)	0.010 (2.56)	0.028 (7.08)
4.5; −3	0.383 ± 0.027	0.382 ± 0.024	0.85 (0.74–0.91)	0.011 (2.90)	0.031 (8.05)
4.5; −6	0.383 ± 0.029	0.380 ± 0.027	0.87 (0.78–0.93)	0.011 (2.79)	0.030 (7.72)
4.5; 3	0.389 ± 0.029	0.390 ± 0.028	0.83 (0.72–0.90)	0.011 (2.74)	0.029 (7.59)
4.5; 6	0.387 ± 0.030	0.388 ± 0.029	0.87 (0.78–0.92)	0.010 (2.69)	0.028 (7.46)
6; 0	0.362 ± 0.027	0.362 ± 0.025	0.84 (0.73–0.90)	0.011 (2.94)	0.030 (8.15)
9; 0	0.415 ± 0.038	0.413 ± 0.034	0.82 (0.71–0.90)	0.015 (3.64)	0.042 (10.10)
Stride time (s)					
3; 0	1.279 ± 0.096	1.300 ± 0.088	0.83 (0.67–0.90)	0.036 (2.81)	0.100 (7.79)
4.5; 0	1.085 ± 0.059	1.091 ± 0.056	0.93 (0.88–0.96)	0.015 (1.35)	0.041 (3.75)
4.5; −3	1.069 ± 0.058	1.068 ± 0.048	0.91 (0.84–0.95)	0.019 (1.73)	0.053 (4.79)
4.5; −6	1.052 ± 0.064	1.049 ± 0.056	0.91 (0.84–0.95)	0.019 (1.73)	0.053 (4.80)
4.5; 3	1.100 ± 0.065	1.103 ± 0.059	0.87 (0.77–0.92)	0.020 (1.83)	0.054 (5.07)
4.5; 6	1.104 ± 0.065	1.108 ± 0.061	0.90 (0.83–0.94)	0.019 (1.80)	0.052 (4.99)
6; 0	0.979 ± 0.053	0.981 ± 0.050	0.95 (0.91–0.97)	0.012 (1.24)	0.034 (3.44)
9; 0	0.758 ± 0.044	0.757 ± 0.043	0.94 (0.89–0.96)	0.011 (1.47)	0.031 (4.07)
Stride length (m)					
3; 0	1.071 ± 0.085	1.090 ± 0.076	0.82 (0.67–0.90)	0.033 (3.01)	0.090 (8.34)
4.5; 0	1.352 ± 0.080	1.359 ± 0.072	0.91 (0.85–0.95)	0.022 (1.63)	0.061 (4.53)
4.5; −3	1.328 ± 0.077	1.323 ± 0.059	0.89 (0.82–0.94)	0.027 (1.93)	0.074 (5.34)
4.5; −6	1.296 ± 0.082	1.289 ± 0.074	0.90 (0.84–0.95)	0.026 (1.87)	0.072 (5.19)
4.5; 3	1.376 ± 0.085	1.378 ± 0.076	0.84 (0.73–0.91)	0.028 (2.08)	0.077 (5.77)
4.5; 6	1.376 ± 0.088	1.382 ± 0.079	0.89 (0.82–0.94)	0.025 (1.96)	0.070 (5.42)
6; 0	1.620 ± 0.093	1.614 ± 0.090	0.86 (0.77–0.92)	0.034 (2.10)	0.094 (5.82)
9; 0	1.831 ± 0.128	1.821 ± 0.125	0.93 (0.88–0.96)	0.033 (1.83)	0.092 (5.06)
Single support time (s)					
3; 0	0.439 ± 0.040	0.443 ± 0.040	0.82 (0.697–0.89)	0.017 (3.85)	0.047 (10.67)
4.5; 0	0.387 ± 0.026	0.390 ± 0.027	0.86 (0.757–0.92)	0.010 (2.56)	0.028 (7.14)
4.5; −3	0.383 ± 0.027	0.382 ± 0.024	0.85 (0.741–0.91)	0.011 (2.90)	0.031 (8.04)
4.5; −6	0.383 ± 0.029	0.380 ± 0.027	0.87 (0.780–0.93)	0.011 (2.78)	0.030 (7.71)
4.5; 3	0.389 ± 0.029	0.390 ± 0.028	0.84 (0.720–0.90)	0.011 (2.75)	0.029 (7.61)
4.5; 6	0.386 ± 0.030	0.388 ± 0.029	0.87 (0.774–0.92)	0.010 (2.70)	0.029 (7.48)
6; 0	0.362 ± 0.027	0.362 ± 0.025	0.84 (0.724–0.90)	0.011 (2.96)	0.030 (8.20)
9; 0	0.327 ± 0.031	0.327 ± 0.030	0.82 (0.700–0.89)	0.013 (3.99)	0.036 (11.05)
Double support time (s)					
3; 0	0.401 ± 0.052	0.414 ± 0.046	0.79 (0.62–0.89)	0.021 (5.12)	0.058 (14.18)
4.5; 0	0.310 ± 0.041	0.311 ± 0.036	0.76 (0.60–0.86)	0.019 (6.15)	0.053 (17.05)
4.5; −3	0.303 ± 0.046	0.304 ± 0.038	0.78 (0.64–0.87)	0.021 (6.60)	0.059 (18.30)
4.5; −6	0.286 ± 0.046	0.289 ± 0.040	0.82 (0.70–0.90)	0.019 (5.78)	0.053 (16.01)
4.5; 3	0.323 ± 0.048	0.323 ± 0.042	0.82 (0.69–0.89)	0.018 (5.99)	0.050 (16.61)
4.5; 6	0.331 ± 0.047	0.332 ± 0.042	0.86 (0.77–0.92)	0.016 (5.54)	0.044 (15.37)
6; 0	0.255 ± 0.039	0.257 ± 0.037	0.80 (0.67–0.88)	0.017 (6.63)	0.047 (18.36)
Foot ground angle at initial contact (°)					
3; 0	17.050 ± 3.657	17.503 ± 3.305	0.76 (0.61–0.86)	1.693 (9.80)	4.692 (27.15)
4.5; 0	22.694 ± 3.691	22.688 ± 3.552	0.71 (0.53–0.83)	1.973 (8.67)	5.469 (24.03)
4.5; −3	25.102 ± 4.315	24.505 ± 3.777	0.82 (0.70–0.90)	1.676 (8.64)	4.645 (23.95)
4.5; −6	27.738 ± 4.668	26.936 ± 4.032	0.85 (0.75–0.92)	1.525 (9.70)	4.226 (26.89)
4.5; 3	19.352 ± 3.952	19.383 ± 3.940	0.80 (0.67–0.88)	1.793 (7.23)	4.969 (20.03)
4.5; 6	15.706 ± 4.062	15.746 ± 3.774	0.81 (0.68–0.89)	1.847 (6.77)	5.119 (18.76)
6; 0	28.147 ± 4.173	27.652 ± 3.905	0.81 (0.69–0.89)	1.734 (6.22)	4.807 (17.23)
9; 0	20.173 ± 7.101	20.026 ± 6.053	0.80 (0.67–0.88)	2.979 (14.82)	8.257 (41.08)
Foot ground angle at toe off (°)					
3; 0	49.360 ± 5.189	50.249 ± 5.374	0.85 (0.74–0.92)	1.969 (3.96)	5.457 (10.97)
4.5; 0	60.295 ± 5.098	60.055 ± 5.056	0.86 (0.76–0.92)	1.916 (3.18)	5.310 (8.82)
4.5; −3	56.937 ± 5.135	57.602 ± 4.929	0.85 (0.75–0.91)	2.081 (3.33)	5.769 (9.23)
4.5; −6	53.158 ± 5.328	53.808 ± 5.429	0.83 (0.72–0.90)	2.222 (3.45)	6.160 (9.57)
4.5; 3	62.387 ± 5.332	62.572 ± 5.369	0.80 (0.67–0.89)	2.210 (3.86)	6.125 (10.69)
4.5; 6	64.361 ± 5.374	64.394 ± 5.346	0.83 (0.72–0.90)	2.195 (4.10)	6.086 (11.37)
6; 0	68.512 ± 5.249	68.028 ± 5.314	0.81 (0.69–0.89)	2.284 (3.35)	6.330 (9.27)
9; 0	54.712 ± 7.440	55.825 ± 7.675	0.77 (0.62–0.86)	3.615 (6.54)	10.020 (18.13)

Abbreviations: ICC 2,1, intraclass correlation coefficient model 2,1; MDC, minimal detectable change; SD, standard deviation; SEM, standard error of measurement.

The ICC values for the spatiotemporal variables ranged from good to excellent across all conditions. Stride time showed the highest reliability, with ICC values exceeding 0.90 in most conditions, indicating excellent reliability. Similarly, swing time, stance time and stride length demonstrated good to excellent reliability across conditions, with ICC values predominantly above 0.85. The lowest ICC values were observed for the kinematic variables, particularly FGA at IC at 4.5 km/h without slope, where the ICC reached 0.71, the only value classified as moderate. FGA at TO showed slightly higher, but still lower reliability than spatiotemporal variables.

SEM% values were low (around 1%–4%) for all spatiotemporal variables (except for double support time, around 5%–7%) across all conditions, reflecting minimal measurement error and suggesting stable measurement precision. SEM% values were higher for kinematic variables (still mainly <10%; indicating low measurement error), except for FGA at IC during running (14.820%; indicating high measurement error). Test conditions had minimal impact on SEM% values. Only during slow walking (3 km/h) and running (9 km/h) were slightly increased SEM% values observed for most variables, indicating a minor reduction in measurement precision under these conditions.

MDC values followed a similar pattern. Most spatiotemporal variables showed MDC% values categorised as low, with most remaining below 10%, indicating good measurement consistency. Double support time showed MDC% values at the higher end of the low category (often between 15% and 20%). Kinematic variables displayed greater variability, with MDC% for FGA at IC categorised as acceptable or high (during running). Test conditions had minimal impact on MDC% values, except during slow walking (3 km/h) and running (9 km/h), where slightly higher MDC% values were observed for most variables, reflecting a slight increase in measurement variability under these conditions.

**Figure 3:**
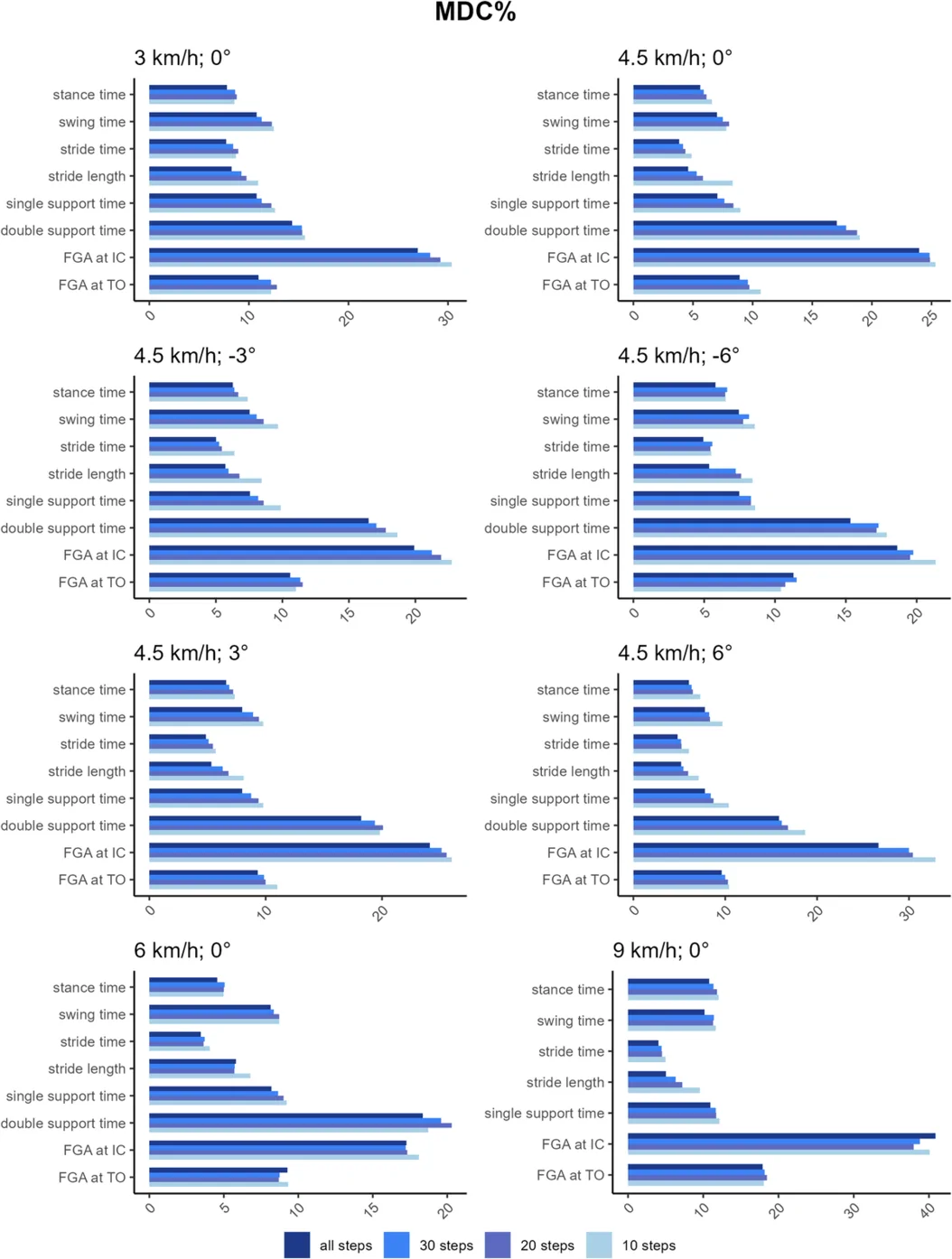
Sensitivity analyses of MDC% (*x*‐axis) across different step counts for all variables and conditions. FGA, foot ground angle; IC, initial contact; MDC, minimal detectable change; TO, toe‐off.

Sensitivity analysis (Figure 3) revealed that, in most conditions, reducing the number of steps included in the analysis only slightly increased MDC% values (difference ranging from 0% to 3%). These results indicate that although the number of steps has some influence on the MDC%, the effect is marginal, and good reliability can already be obtained with 10 steps for most variables investigated.

## DISCUSSION

The aim of this study was to assess the test–retest reliability of the WSS for quantifying spatiotemporal and kinematic gait variables in healthy adults across various walking and running conditions. In total, 49 healthy participants completed walking and running trials at different speeds and slope conditions on two separate occasions, allowing for a comprehensive evaluation of the device's measurement consistency.

The current study demonstrates good to excellent reliability of the WSS for most spatiotemporal gait variables, as reflected by ICC values exceeding 0.90 for stride time, and generally above 0.85 for swing time, stance time and stride length. These findings are consistent with test–retest values reported for similar insole‐based systems, such as the Moticon OpenGo and Insole 3 systems, where ICCs typically range from 0.97 to 0.99 [3, 6] and from 0.80 to 0.99 for PODOSmart® [25]. However, some key methodological differences should be considered when comparing these findings. For example, Braun et al. [3] conducted repeated measurements within the same session, which eliminates potential variability due to sensor repositioning or daily fluctuations. In contrast, the present study applied a test–retest interval of one week, providing a more realistic evaluation of the system's reliability under typical use conditions. Furthermore, both Braun et al. [3] and Cramer et al. [6] included relatively small samples (*N* = 11–12) and tested only a limited range of walking conditions. In contrast, the current study included a larger sample size and assessed multiple walking conditions, thereby enhancing the generalizability and practical relevance of the results. According to the interpretation guidelines by Koo and Li [20], these ICC values indicate a high level of agreement, supporting the device's potential for reliable use in clinical or research settings.

The double support time showed slightly lower but still good reliability (ICC > 0.75), consistent with previous findings where time‐based measures such as double support time also showed moderate to good reliability [12, 33]. It is important to note that Hansen et al. [12] employed a single IMU placed on the lower back, representing a system without direct insole data. In contrast, Riglet et al. [33] used instrumented insoles (DSPro®) exclusively, reporting good reliability for double support time (ICC > 0.8). The WSS integrates both IMUs and instrumented insoles. However, double support time measurements within WSS are derived solely from the insole sensors (see Eckelt et al. [9] for detailed methodology). These sensors detect foot contact events directly, making the double support time measurement in the WSS conceptually and methodologically comparable to the values reported by Riglet et al. [33] for DSPro®. To further contextualise these findings, the MDC values observed in the present study (0.044–0.059 s; MDC%: 14–18) indicate the smallest change in double support time that is likely a true change rather than just random measurement error in healthy adults. Thus, MDC values can help assess whether clinically relevant changes fall within the detectable range of the WSS. A recent meta‐analysis in patients following total knee arthroplasty reported typical improvements in double support time of approximately 0.05 s (WMD = −0.05; 95% CI: −0.02 to −0.08) [40], suggesting that the WSS would be capable of detecting changes of this magnitude. However, it is important to note that the current study assesses reliability rather than clinical responsiveness, and direct comparisons should be interpreted with caution.

Kinematic variables, particularly the FGA at IC, displayed greater variability, with ICC values falling into the moderate range at the walking speed of 4.5 km/h, with no slope. This aligns with previous research suggesting that kinematic variables derived from wearable insoles may be more sensitive to sensor placement and individual gait adaptations [14, 33]. For instance, Riglet and colleagues [33] reported lower ICC values for some kinematic parameters compared to spatiotemporal variables when using embedded IMU insoles, highlighting their greater measurement variability and the need for cautious interpretation.

The SEM and MDC values provide additional insight into the precision and sensitivity of the device. SEM values were low for most spatiotemporal variables with SEM%, generally below 5% (except for double support time), indicating a stable measurement error across test and retest sessions. Similarly, MDC values were consistently within the low category, typically below 10%, suggesting that the system is capable of detecting relatively small changes in gait. SEM% values below 5% and MDC values below 10% are frequently regarded in clinical and rehabilitation settings as indicative of adequate measurement precision and potentially meaningful change [8]. These threshold values fall within or below the ranges reported for other wearable insoles, where, for instance, MDCs for variables such as step time, stride time and swing/stance time were between 12 and 22% [12, 21].

However, MDC% values for kinematic variables were substantially higher than for the spatiotemporal variables, in particular for the FGA at IC, which exceeded 20% in most conditions. This reflects a higher degree of measurement variability and indicates that changes in these angular variables should be interpreted with caution. This finding is in line with earlier reports suggesting that FGA‐related variables may require further validation to ensure sufficient reliability for clinical use [38].

Our findings thus confirm the initial hypothesis that reliability is higher for spatiotemporal variables compared to kinematic variables. This is consistent with previous reports highlighting the excellent accuracy and repeatability of spatiotemporal gait measures obtained using wearable insole‐based systems [15, 16, 23]. It is important to note that, in the present study, both spatiotemporal and kinematic variables were derived from the same wearable system that integrates insole and IMU data. This ensures that differences in reliability between parameter types cannot be attributed to differences in sensor technology. The superior reliability of spatiotemporal variables is likely due to their derivation from discrete gait events, such as IC and TO, which can be detected with high precision using the combined system. In contrast, the calculation of kinematic variables involves sensor fusion from both insoles and IMUs, introducing additional processing steps and potential sources of error, which may explain their lower reliability. Previous studies have raised concerns about IMU systems mounted on shoes, as sensor movement relative to the shoe can compromise measurement accuracy [14, 33]. Furthermore, kinematic variables are more sensitive to soft tissue artefacts, sensor drift and individual adaptations in foot motion [19, 32]. Although the system demonstrated excellent reliability for most conditions, the consistency of kinematic measurements—particularly FGA—was lower. This may reflect sensor positioning errors or limitations in signal processing. Future refinements in sensor placement standardisation, calibration procedures and algorithmic filtering could help enhance the reliability of these variables in clinical and research settings. Together, these factors likely explain the higher variability and lower reliability observed for kinematic compared to spatiotemporal variables in our study.

Interestingly, the sensitivity analysis using different step counts revealed that reducing the number of analysed steps only slightly affected MDC% values, with changes generally within a narrow range of 2%–3%. These results support the feasibility of using shorter walking bouts in certain clinical or time‐constrained scenarios, although caution is warranted, as the reliability may vary depending on the specific parameter and walking condition being analysed.

While the present study provides comprehensive insights into the reliability of the WSS for gait analysis under diverse conditions, several limitations should be considered when interpreting the findings. First, the study sample consisted solely of healthy young adults, which limits generalisability to clinical populations such as older adults, individuals with neurological or musculoskeletal impairments, or children. Gait variability and test–retest reliability may differ significantly in populations with unstable or pathological gait patterns, where wearable systems may perform differently [35, 38]. Second, although a broad range of walking and running speeds and slopes was tested, the analysis was restricted to treadmill‐based assessments. As treadmill gait can differ biomechanically from overground walking [22], future studies should evaluate the system's reliability in real‐world, overground environments where surface irregularities and turning behaviour may influence sensor accuracy. Nevertheless, the treadmill approach was deliberately chosen to enable continuous, controlled measurements over one minute per condition while minimising external noise sources. Third, the study focused on a selected set of conventional spatiotemporal variables and kinematic angles, leaving out other potentially informative gait parameters. As the field of quantitative gait analysis continues to evolve, additional measures such as gait symmetry, variability, or kinetic data may offer valuable insights and warrant investigation in future work [12]. The variables analysed in this study were chosen because they are widely used as reference values for detecting gait abnormalities [13, 17]. Lastly, the reference for reliability was the agreement between two measurements collected one week apart. Although this interval is suitable for test–retest reliability [20], it does not account for intra‐day variability or longer‐term stability, both of which are relevant for monitoring interventions or disease progression.

## CONCLUSION

This study demonstrated that the WSS provides reliable data for spatiotemporal gait variables across various walking and running conditions. However, kinematic measurements (FGA at IC and TO) exhibited greater variability, suggesting that further investigation into the reliability of angular variables is needed. These findings highlight the potential of the WSS for real‐world gait assessment, while identifying areas for improvement. Future research should also focus on the applicability of the WSS in clinical settings and in populations with atypical gait patterns.

## AUTHOR CONTRIBUTIONS

All authors contributed to the study conception and design. Material preparation, data collection and analysis were performed by Jennifer Fayad, Anne Backes, Valeria Serchi and Melanie Eckelt. The first draft of the manuscript was written by Melanie Eckelt, and all authors commented on previous versions of the manuscript. All authors read and approved the final manuscript.

## CONFLICT OF INTEREST STATEMENT

The authors declare that Tobias Meyer, Dr. Valeria Serchi and Thomas Solignac are employees of IEE S.A., the manufacturer of the insole investigated in this study. All conflicts of interest have been disclosed in accordance with the journal's guidelines and did not influence the design, execution, analysis, or interpretation of the research. The remaining authors declare no conflict of interest.

## ETHICS STATEMENT

This study was conducted in accordance with the ethical standards of the Declaration of Helsinki and was approved by the National Ethics Committee for Research (CNER) (Reference: 202212/04 Version 2.0). All participants provided written informed consent prior to participation.

## Supporting information

WSS Reliability Supplementary file.

## Data Availability

The datasets generated during the current study are not publicly available but are available from the corresponding author on reasonable request.
