# Supplementary material for: Reliability of a wireless instrumented insole (WalkinSense system) for measuring spatiotemporal and kinematic gait variables
Source: J Exp Orthop. 2026 Jan 22;13(1):e70628. doi: 10.1002/jeo2.70628 (PMC12825025; doi:10.1002/jeo2.70628)
Supplement: Supplementary file 1 — WSS Reliability Supplementary file. [file JEO2-13-e70628-s001.docx]

Supplementary File

Bland-Altman analyses for all gait variables at different test conditions


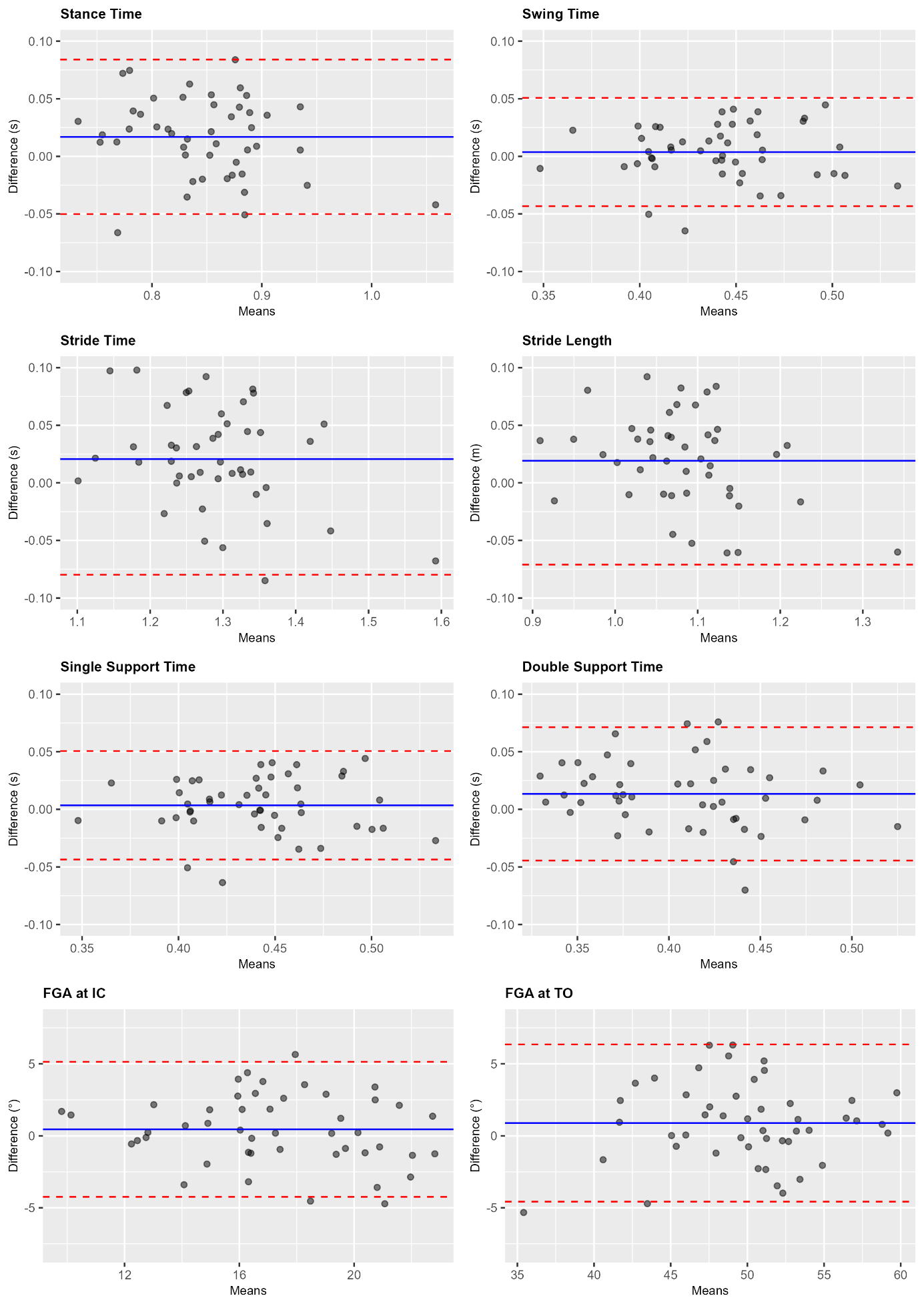


Figure S1: Bland-Altman Plots for different gait metrics at 3 km/h (no slope)


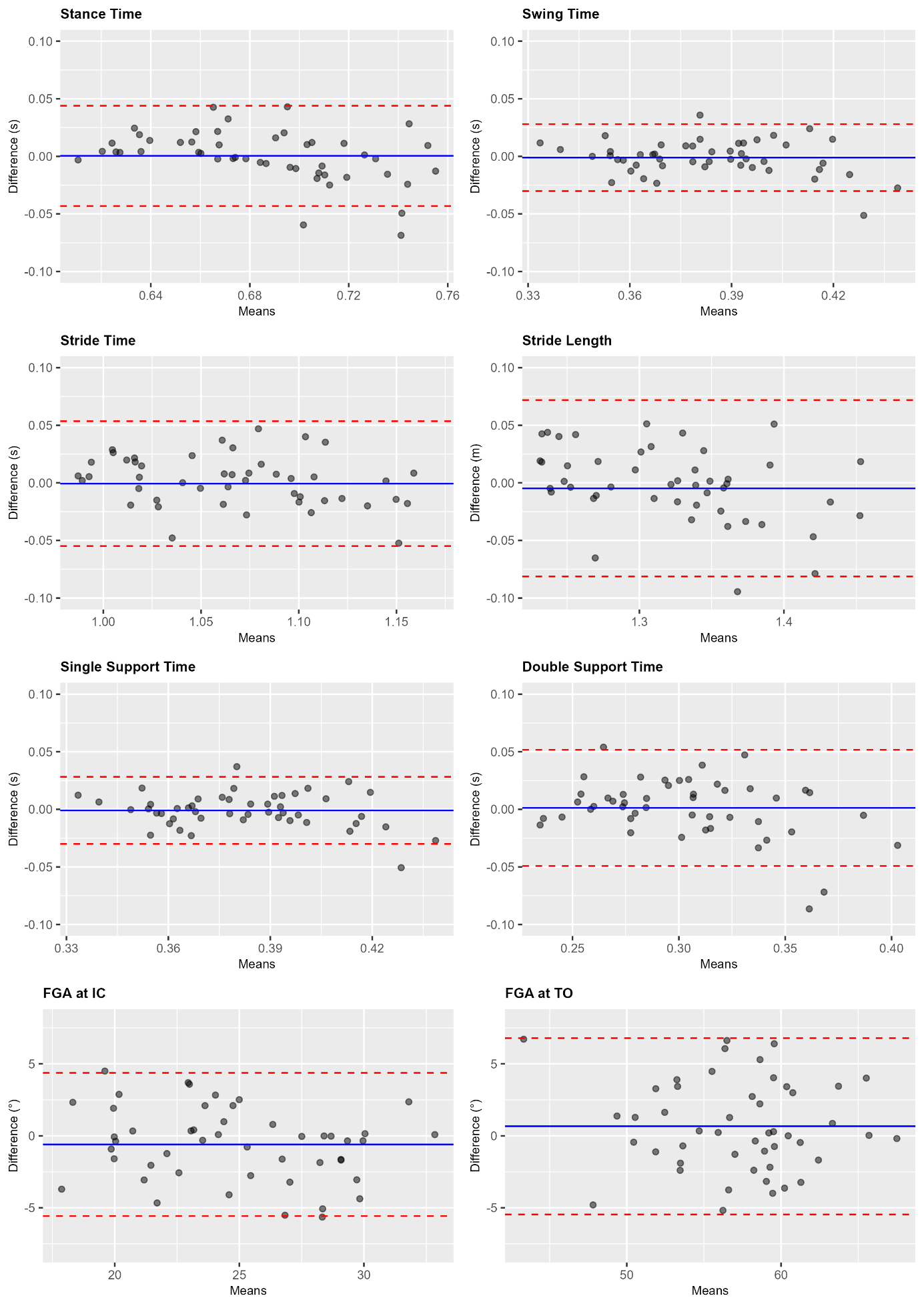


Figure S2: Bland-Altman Plots for different gait metrics at 4.5 km/h (-3° slope)


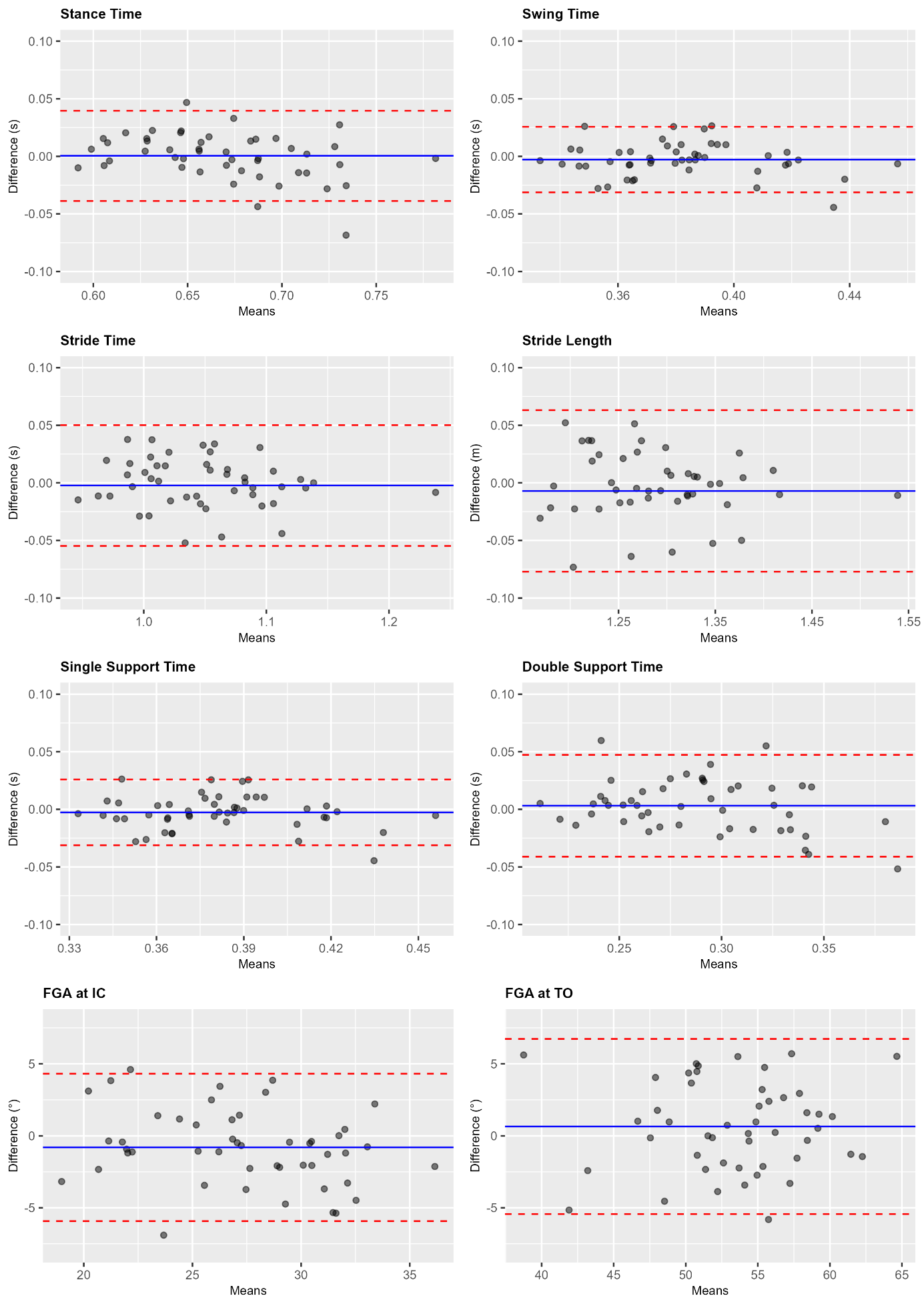


Figure S3: Bland-Altman Plots for different gait metrics at 4.5 km/h (-6° slope)


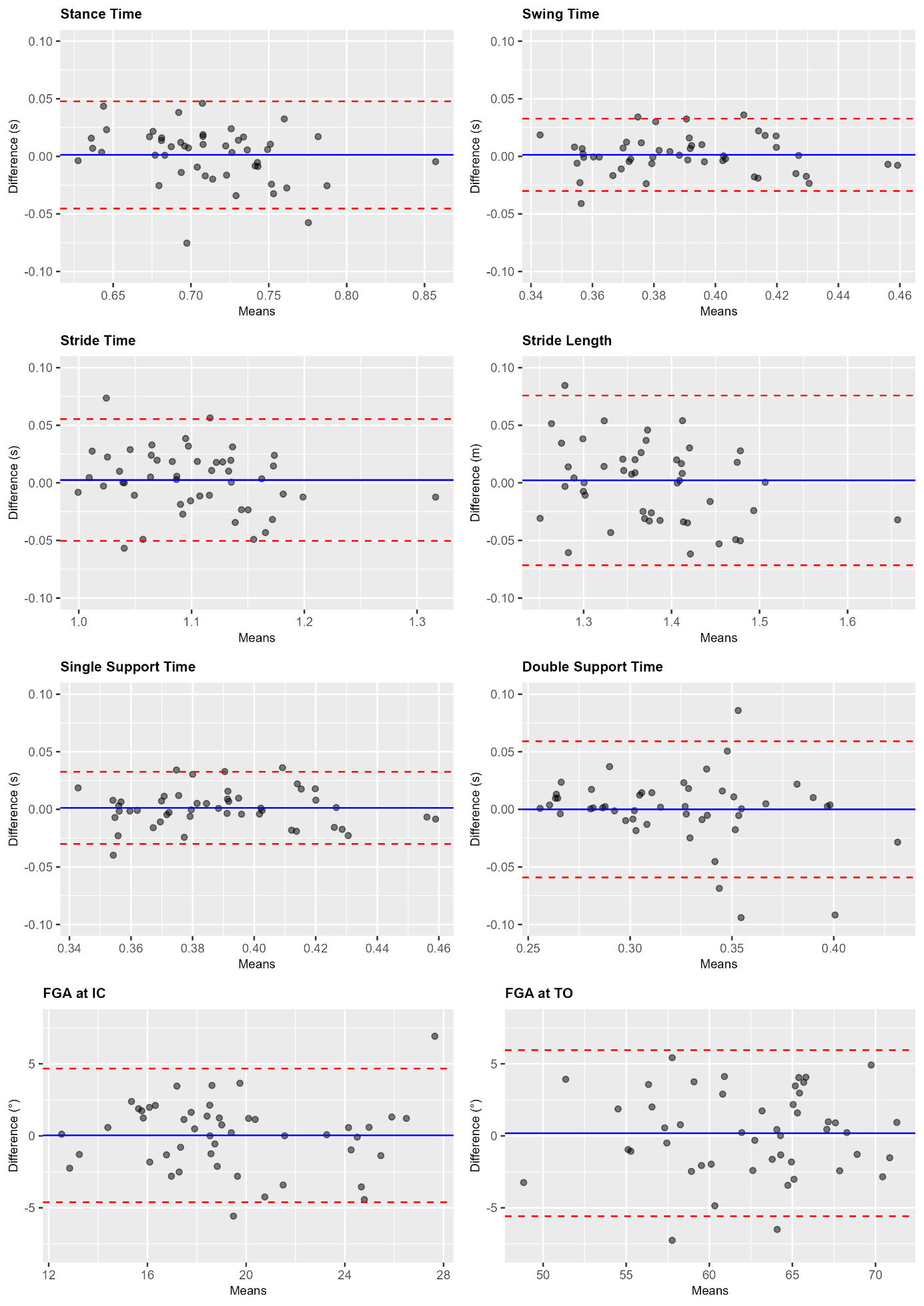


Figure S4: Bland-Altman Plots for different gait metrics at 4.5 km/h (3° slope)


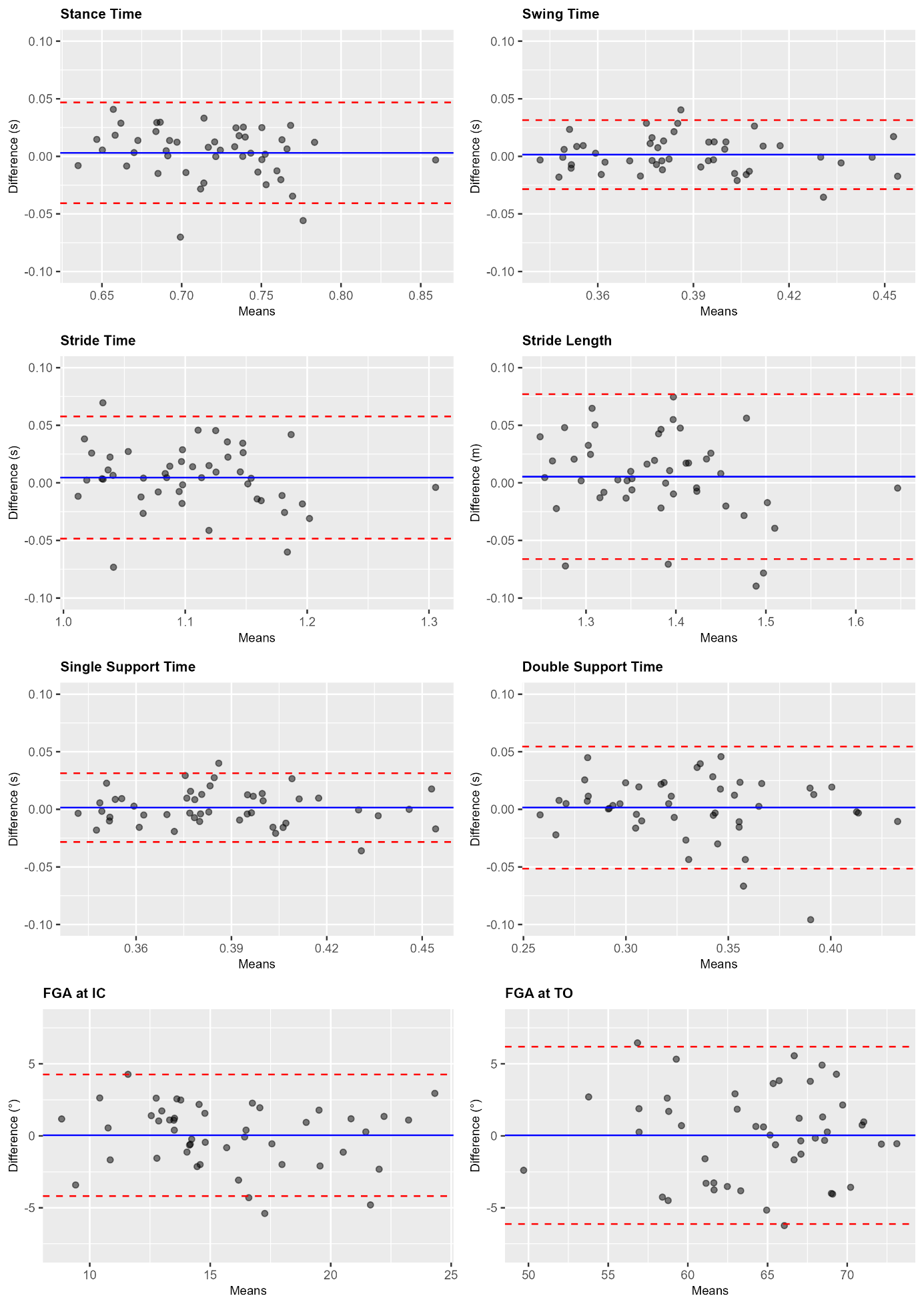


Figure S5: Bland-Altman Plots for different gait metrics at 4.5 km/h (6° slope)


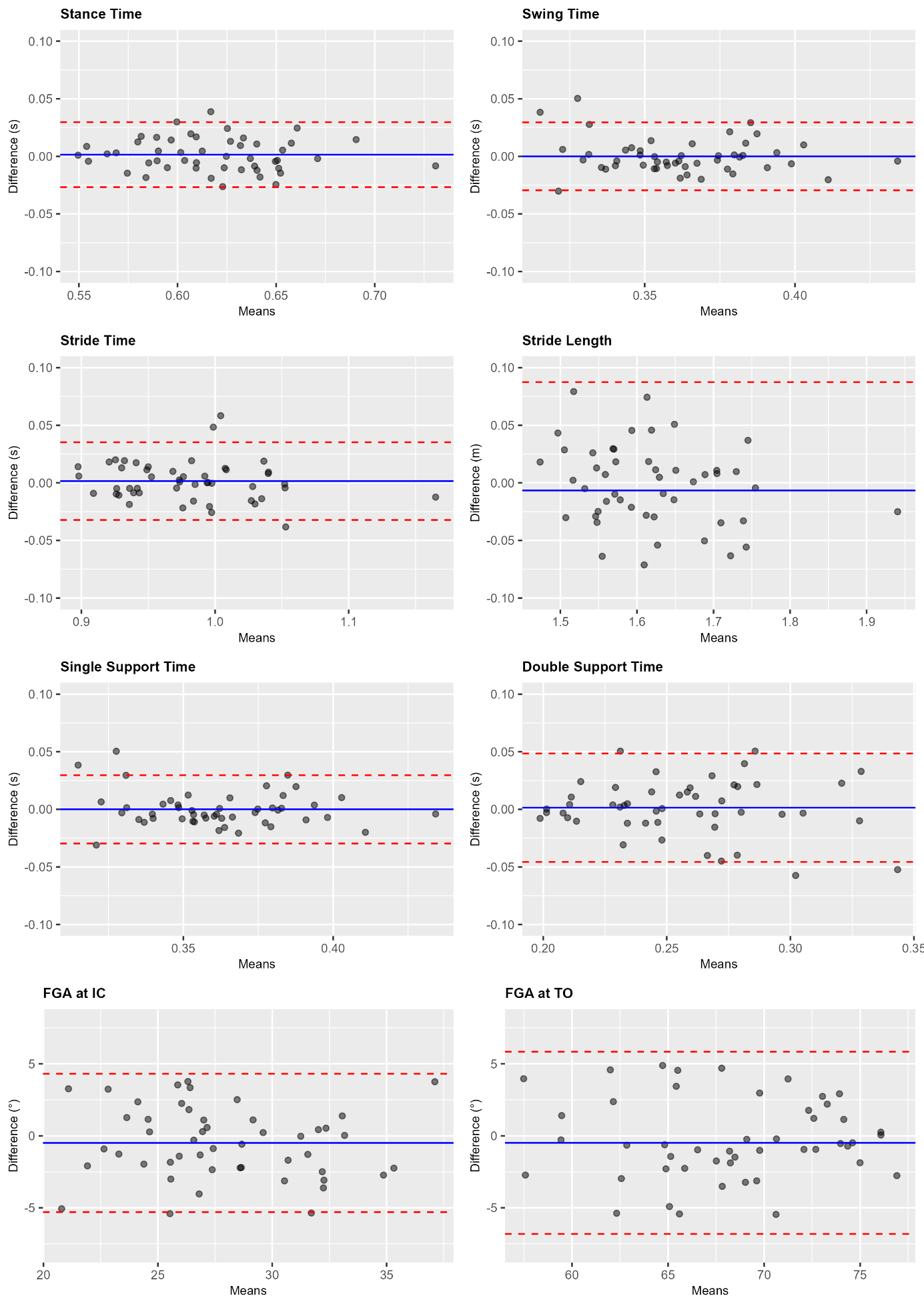


Figure S6: Bland-Altman Plots for different gait metrics at 6 km/h (no slope)


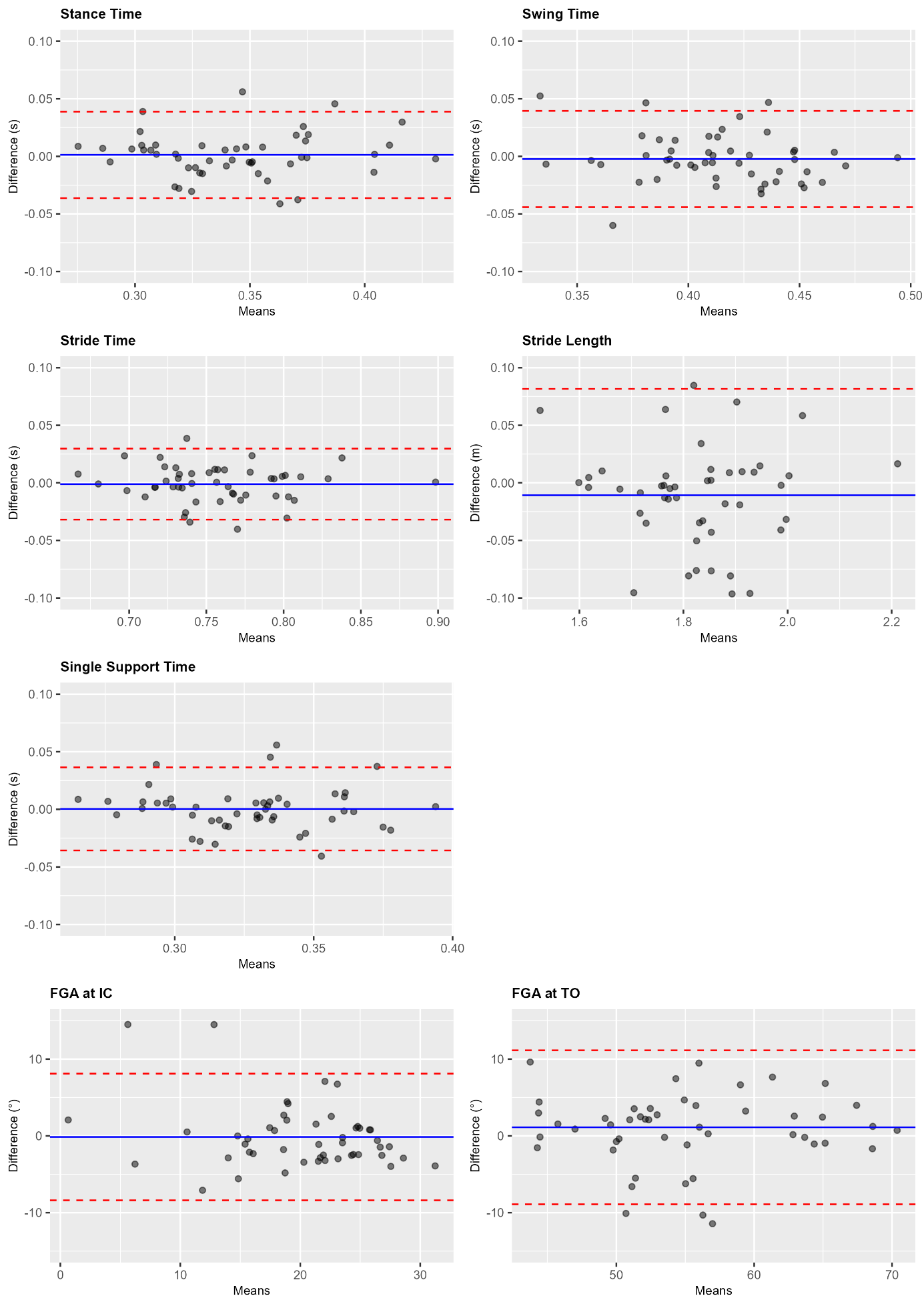


Figure S7: Bland-Altman Plots for different gait metrics at 9 km/h (no slope)
